# Transcriptome Analysis of Leaves, Flowers and Fruits Perisperm of *Coffea arabica* L. Reveals the Differential Expression of Genes Involved in Raffinose Biosynthesis

**DOI:** 10.1371/journal.pone.0169595

**Published:** 2017-01-09

**Authors:** Suzana Tiemi Ivamoto, Osvaldo Reis, Douglas Silva Domingues, Tiago Benedito dos Santos, Fernanda Freitas de Oliveira, David Pot, Thierry Leroy, Luiz Gonzaga Esteves Vieira, Marcelo Falsarella Carazzolle, Gonçalo Amarante Guimarães Pereira, Luiz Filipe Protasio Pereira

**Affiliations:** 1 Programa de Pós-Graduação em Genética e Biologia Molecular, Centro de Ciências Biológicas, Universidade Estadual de Londrina (UEL), Londrina, Brazil; 2 Laboratório de Biotecnologia Vegetal, Instituto Agronômico do Paraná (IAPAR), Londrina, Brazil; 3 Laboratório de Genômica e Expressão, Departamento de Genética, Evolução e Bioagentes, Instituto de Biologia, Universidade Estadual de Campinas (UNICAMP), Campinas, Brazil; 4 Departamento de Botânica, Instituto de Biociências de Rio Claro, Universidade Estadual Paulista (UNESP), Rio Claro, Brazil; 5 Centre de Coopération Internationale en Recherche Agronomique Pour le Développement, (CIRAD), UMR AGAP, Montpellier, France; 6 Programa de Pós Graduação em Agronomia, Universidade do Oeste Paulista (UNOESTE), Presidente Prudente, Brazil; 7 Empresa Brasileira de Pesquisa Agropecuária (Embrapa Café), Brasília, Brazil; National Institutes of Health, UNITED STATES

## Abstract

*Coffea arabica* L. is an important crop in several developing countries. Despite its economic importance, minimal transcriptome data are available for fruit tissues, especially during fruit development where several compounds related to coffee quality are produced. To understand the molecular aspects related to coffee fruit and grain development, we report a large-scale transcriptome analysis of leaf, flower and perisperm fruit tissue development. Illumina sequencing yielded 41,881,572 high-quality filtered reads. *De novo* assembly generated 65,364 unigenes with an average length of 1,264 bp. A total of 24,548 unigenes were annotated as protein coding genes, including 12,560 full-length sequences. In the annotation process, we identified nine candidate genes related to the biosynthesis of raffinose family oligossacarides (RFOs). These sugars confer osmoprotection and are accumulated during initial fruit development. Four genes from this pathway had their transcriptional pattern validated by quantitative reverse transcription polymerase chain reaction (qRT-PCR). Furthermore, we identified ~24,000 putative target sites for microRNAs (miRNAs) and 134 putative transcriptionally active transposable elements (TE) sequences in our dataset. This *C*. *arabica* transcriptomic atlas provides an important step for identifying candidate genes related to several coffee metabolic pathways, especially those related to fruit chemical composition and therefore beverage quality. Our results are the starting point for enhancing our knowledge about the coffee genes that are transcribed during the flowering and initial fruit development stages.

## Introduction

Coffee represents one of the most important crops in tropical developing countries. The genus has 124 species [1], but only the allotetraploid *Coffea arabica* L. and the diploid *Coffea canephora* Pierre ex A. Froehner have economic importance, accounting for approximately 70% and 30% of world coffee production, respectively [2]. Despite its economic importance, the *C*. *arabica* genome has not been published to date, and only the genome of one *C*. *arabica* diploid ancestor, *C*. *canephora*, was recently published [3]. Coffee transcriptome studies have been perfomed [4–8] but so far, very few data is available for *C*. *arabica* flower and fruit development.

RNA-seq is considered a powerful molecular tool for investigating non-model species that have little information available for genetic studies [9]. The identification of candidate genes related to agronomic traits and their transcriptional profile might reveal new hypotheses about genetic mechanisms that control proteins and metabolites biosynthesis. Currently, high-throughput mRNA sequencing techniques (RNA-seq) have been widely used in studies of plant transcriptomes.

The transcriptome can also contain non-coding RNAs and other genomic components. In plants, microRNAs (miRNAs) play an important role in different biological and metabolic process, including tissue differentiation and development, signal transduction, response to abiotic/biotic stresses conditions and fruit development [10–11]. In addition, transposable elements (TE), which are major components of plant genomes, might also shape the architecture, function and expression of plant genes and genomes throughout evolution [12]. In coffee plants, albeit previous studies have shown low TE expression, they can be detected in transcriptome analyses [13–14].

Coffee beverage is obtained from ground seed endosperm; however, most RNA-seq public data represents the leaf transcriptome. Among the 42 transcriptome analyses of *C*. *arabica* deposited in the Short Read Archive (SRA) of NCBI by August 2016, no study has addressed transcriptional profiles in flowers or fruit tissues. In coffee, most of the metabolites in the fruits are synthetized during the development of perisperm. Perisperm is a highly active tissue with an intense metabolism and is replaced by endosperm during fruit development [15–16].

The accumulation of raffinose family oligosaccharides (RFOs), such as raffinose and stachyose, was previously observed during coffee fruit development [17–18]. RFOs are compatible solutes that are typically involved in stress tolerance defense mechanisms. RFOs act as signal molecules in response to stress [19–20] and are related to seed desiccation tolerance and germination [21–22]. In coffee plants, RFOs are involved in osmoprotection against abiotic stresses in leaves [23–24], but they can also be possible donors of carbon skeletons during the synthesis of cell wall storage polysaccharides (CWSPs). A microarray-based analysis in coffee endosperm showed that the *GolS* transcript levels were significantly correlated with the amount of CWSPs [17].

In this study, we analyzed *de novo* assembled transcriptome data from leaves, flowers and coffee fruit perisperm in five development stages and identified genes that are specifically expressed in these organs. We also generated a catalog of putative transcriptionally active transposable elements and miRNA targets, which are relevant transcriptome components that are rarely studied using transcriptomic approaches. Genes related to RFOs biosynthesis had their transcriptional pattern confirmed by qRT-PCR, which suggests that our large-scale transcriptome resources will add valuable information for the discovery of key genes involved in coffee fruit metabolism.

## Materials and Methods

### Plant materials

Tissues were obtained from 20-year-old individual *C*. *arabica* cv. IAPAR59 plants grown at the Agronomic Institute of Paraná (Londrina—Brazil) under full-sun field conditions with standard irrigation and fertilization practices. We collected leaves (3^rd^ pair from plagiotropic branches in the middle third of the tree), open flowers and fruits. The fruit samples were harvested monthly after flowering (30 to 150 DAF; from October 2011 to May 2012). Fruit tissues were separated into pulp, perisperm and endosperm, and only perisperm was selected for RNA sequencing. All samples were collected between 9 and 11 a.m., transferred immediately to liquid nitrogen and stored at -80°C until RNA extraction.

### RNA extraction

Plant materials were pulverized in liquid nitrogen using a cooled mortar and pestle. Total RNA was isolated based on the method of Chang et al. (1993) [25]. The integrity of the RNA samples was examined by 1% agarose gel electrophoresis, and the samples were treated with DNase (RNase-free) to remove genomic DNA contamination. The quality and concentration of extracted RNAs were verified using a NanoDrop^®^ ND-1000 spectrophotometer (Thermo Scientific, Wilmington, DE, USA) and confirmed using a Bioanalyzer Chip DNA 1000 series II (Agilent, Santa Clara, CA, USA).

### RNA sequencing

The mRNA sequencing was performed at the High-Throughput Sequencing Facility at the Carolina Center for Genome Sciences (University of North Carolina, Chapel Hill, NC, USA). For each sample, 10 μg of total RNA was used to prepare mRNA libraries for sequencing and we followed Illumina standard protocol. Library quality control and quantification were performed using a Bioanalyzer Chip DNA 1000 series II (Agilent, Santa Clara, CA, USA). All libraries were tagged and multiplexed in Illumina HiSeq™ 2000, generating 100-base-pair (bp) single-end sequences. RNA-seq data were submitted to NCBI under BioProject accession number PRJNA339585. Transcriptome Sequencing Analysis (TSA) and Sequence Read Arquive (SRA) files are available under GEXP00000000 and SRP082511 accession numbers, respectively.

### RNA-seq data processing and de novo assembly

Raw reads from RNA-seq were filtered by discarding read adaptors contamination and low sequencing quality regions using an in house PERL script that excluded sequences with Phred quality below 20. Processed reads of all libraries were merged for assembly using Trinity assembler, 6-8-2012 version [26], using an optimized k-mer length of 25 for *de novo* assembly. Contigs with a minimal length of 200 bp were used for further analyses. Putative coding sequences were predicted using Transdecoder (https://transdecoder.github.io/).

### Transcriptome gene atlas annotation and classification

All unigenes were compared against NCBI non-redundant sequence (nr) and Swiss-Prot database [27] using BlastX, with an e-value cutoff of 1e-5. Comparison analyses of transcriptome unigenes were also performed against *C*. *arabica* public EST assemblies [6], *C*. *canephora* [3] and *C*. *eugenioides* coding sequences [28] with an e-value cutoff of 1e-5. Functional annotation describing biological processes, molecular function and cellular component were performed using Blast2GO v.2.7.0 tools [29]. We also used InterProScan [30] to identify conserved protein domains and KEGG database [31] to identify metabolic pathways that were available in the sequenced transcriptome, both annotation were done using Blast2GO tools using default parameters or as previously described [28].

### Digital gene expression analysis

We used Bowtie [32] with the default parameters to map all of the reads against the *de novo* assembled transcriptome, allowing a maximum of three mismatches. RPKM (reads per kilobase of transcript per million fragments sequenced) values were normalized for each unigene based on the Robinson and Oshlack method [33]. Pairwise comparisons of expression data analysis among leaves and flowers and during the initial perisperm development stages (30 to 150 DAF) were used to identify differentially gene expressed using EdgeR package [34] results. Digital Gene Expression (DGE) analysis among libraries was performed with a cut-off of log2 fold change (Log2FC) ≥ 1 for up-regulated or Log2FC ≤ -1 for down-regulated genes and p ≤ 0.05. Venn diagrams were developed using Calculate and Draw custom Venn Diagrams (http://bioinformatics.psb.ugent.be/webtools/Venn/). Unigenes were annotated using TrapID (Rapid Analysis of Transcriptome Data) platform [35].

### Transposable elements identification

Coffee unigenes were compared against transposable elements sequences available at Repbase protein transposable elements database [36] using a strategy similar to that reported by Santos et al. [37] and Marcon et al. [38]. Unigenes were considered related to TEs when there was a minimum alignment of 200 bp, a score greater than 200 and a 1e-10 evalue in BlastN.

### Prediction of potential conserved miRNAs targets

*Coffea arabica* assembled unigenes were submitted to psRNATarget [39] webserver for predicting miRNA targets. We used the default parameters to identify potential miRNA targets: i) a maximum expectation of 3; ii) a length of 20 for complementarity scoring; iii) a target accessibility, i.e., the allowed maximum energy to unpair the target site (UPE), of 25; iv) a flanking length around target site for target accessibility analysis of 17 bp upstream and 13 bp downstream; and v) a range of central mismatch of 9 to 11 nucleotides leading to translational inhibition.

### Identification of RFO-related genes

Coding sequences of galactinol synthase, raffinose synthase and stachyose synthase genes were obtained from The Arabidopsis Information Resource database (TAIR, www.arabidopsis.org) and used as queries to search by tBlastX their respective orthologs in our coffee transcriptome assembled sequences. Orthologs were assessed by reciprocal best hit (RBH). Enrichment GO analyses using coffee candidate genes related to RFO metabolism were performed using Fisher’s exact test and FDR cutoff of 0.01 developed using the Blast2GO software [29].

### qRT-PCR transcriptional validation

Primers were designed using the Primer 3 software [40] to amplify products ranging from 101 to 105 bp, with a melting temperature of 60°C. Primer sequences are presented in S1 Table. Primer efficiency was calculated using LinRegPCR software [41].

Complementary DNAs (cDNAs) of *C*. *arabica* leaves and perisperm (90, 120 and 150 DAF) were synthesized using a SuperScript III Reverse Transcriptase kit (Invitrogen, Carlsbad, CA, USA), following the manufacturer’s instructions, in a final volume of 20 μl using 5 μg of total RNA. qRT-PCR was performed in a 7500 Fast Real-Time PCR System (Applied Biosystems) and following basic procedures reported a previous publication in coffee plants [42]. The reaction mixture contained 7.5 μl of SYBR Green PCR Master Mix (Applied Biosystems, Foster City, CA, USA), 0.3 μl of each primer (3 μM), 1 μl of cDNA (40 ng/μL) and 5.9 μl of Milli-Q water. The qRT-PCR conditions were 95°C for 5 min; 40 cycles of 94°C for 30 s, 62°C for 60 s, 72°C for 30 s, and a final step of 72°C for 10 min. Melting curves were analyzed to verify the presence of a single product including a negative control. All reactions were performed with three biological and technical replicates, and we followed the MIQE guidelines for qRT-PCR experiments [43].

Relative expression determination and normalization process were developed using the GenEX software (MultiD, Gothenburg, Sweden) with the default parameters. Transcriptional levels were normalized using coffee glyceraldehyde-3-phosphate dehydrogenase *(GAPDH*) and elongation factor 1 (*EF1*) gene expression profiles as references following the previous recommendations for coffee plants [44–45]. Data were analyzed by two-way ANOVA and Tukey’s test (p<0.05) using the Assistat software [46].

## Results

### Transcriptome sequencing and de novo assembly

A total of 41,881,572 high-quality reads were obtained from mRNA sequencing. Because *C*. *arabica* does not have a reference genome, we opted to make a *de novo* assembly where 127,600 contigs were generated. A total of 65,364 transcripts were considered unigenes (unique splicing variants) with size > 200 base pairs (bp), and 24,548 unigenes were predicted as putative proteins with open reading frames. The average length for these 65,364 contigs was 1,264 bp, with a range from 201 to 12,891 bp. We achieved a N50 of 2,118 bp, and the mean GC content was 41.13% (Table 1). Approximately 60% of the contigs had 200 to 500 bp, 16% had 501 to 1,000 bp, 12% had 1,001 to 2,000 bp and 4% were longer than 3,000 bp (S1 Fig).

**Table 1:** Summary of *C*. *arabica de novo* transcriptome assembly.

Assembly Information	Values
High-quality Reads	41,881,572
Percentage of Mapped Reads	65%
GC Content	41,13%
N50	2,118 bp
Total of Contigs	127,600
Number of Unigenes (>200 bp)	65,364
Number of Coding Protein Unigenes	24,548
Number of Full-Length Coding Protein Unigenes	12,560
Unigenes Average Size	1,264 bp

### Transcriptome gene annotation and data mining

Automatic annotation was performed to identify conserved domain sequences and to obtain KEGG metabolic pathways maps to characterize our coffee transcriptome dataset. A total of 24,548 unigenes were successfully annotated as coding protein genes by BlastX, including 12,560 full-length sequences (Table 1).

*Vitis vinifera* (40.64%) was the species with the highest similarity with coffee sequences followed by *Populus trichocarpa* (11.13%), *Ricinus communis* (10.89%) and *Glycine max* (4.24%).

We also investigated the contribution of novel transcripts for coffee transcriptome studies. We compared our assembly with the 35,153 *Coffea arabica* contigs available on CafESTs database [5–6], 25,574 unigenes from the *Coffea canephora* genome [3] and *Coffea eugenioides* transcriptome data (36,935 unigenes) [28]. A total of 26,176 unigenes matched CafEST contigs, 24,798 unigenes matched *C*. *canephora* CDS and 20,542 unigenes matched *C*. *eugenioides* unigenes (Table 2).

**Table 2:** Similarity analysis of coffee and plant database sequences.

Reference Database	Hits	No Hits
*C*. *arabica* ESTs	26,176	39,188
*C*. *canephora* genome	24,798	40,566
*C*. *eugenioides*	20,542	44,822

### Gene ontology analysis

A total of 27,259 molecular functions, 19,373 cellular components and 27,255 biological process terms were associated with our dataset based on the gene ontology (GO) database. The GO classifications were distributed in 15 levels among these three categories. The most informative GO levels for coffee unigenes were five, six and eight, which include a high number of annotated GO terms (S2 Fig). The GO annotation at those intermediary levels allowed inferring putative functions for our unigenes dataset, as we described further down in the RFO gene charatherization.

### Conserved protein domain analysis and KEGG mapping

Using InterProScan, we identified 105,258 conserved domains (CD), representing a total of 5,246 non-redundant CD. The three most abundant terms found were kinases, cytochromes P450 and binding site proteins (S3 Fig).

Subsequently, we mapped unigenes against the KEGG metabolic pathway maps. A total of 130 map pathways were found for the coffee proteins dataset, including 1,484 enzymes for the 5,259 mapped unigenes (24.34%).

### Digital gene expression of *C*. *arabica* unigenes

**Fig 1:**
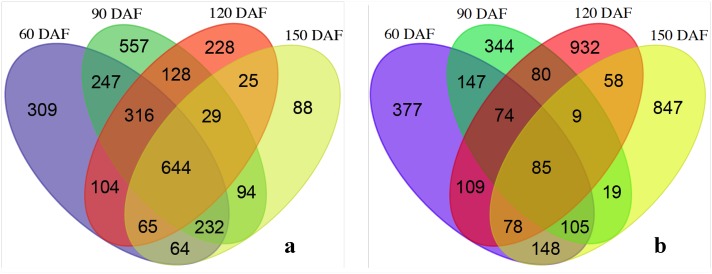
Venn diagrams showing unigenes up- (a) and down-regulated (b) among perisperm development stages (60, 90, 120 and 150 DAF) compared with perisperm at 30 DAF. A total of 3,130 unigenes classified as down-regulated (**a**) and 3,412 unigenes classified as up-regulated (**b**) were used in this analysis.

Digital gene expression (DGEs) analysis using edgeR package was performed to obtain a panel of down- and up-regulated unigenes among the *C*. *arabica* RNA-seq libraries. We performed two DGEs analyses: i) DGEs that were up- and down-regulated among all libraries (Table 3) ii) DGEs that were up- and down-regulated only in perisperm from 30 to 150 DAF (Fig 1a and 1b).

**Table 3:** A summary of up- and down-regulated DGEs among coffee libraries*.

Coffee Libraries	Flower	Leaves	Perisperm				
			30 DAF	60 DAF	90 DAF	120 DAF	150 DAF
**Flower**	-	234	***2115***	288	428	1132	1394
**Leaves**	599	-	***2009***	***130***	363	895	1108
**30 DAF**	1311	680	-	1123	863	1425	1349
**60 DAF**	588	***136***	***1981***	-	614	1558	***3878***
**90 DAF**	919	356	***2247***	799	-	1569	1426
**120 DAF**	828	381	***1539***	1159	790	-	1433
**150 DAF**	822	219	***1241***	1111	568	1232	-

*Up-regulated unigenes are placed at the botton of the table (under—mark), and down-regulated unigenes are placed at the top of the table (above—mark). Number in bold and italic are high and low values, respectively, as observed between their respective libraries. Numbers underlined are the lowest and highest values among all libraries comparisons for up- and down-regulated genes.

After a pairwise analysis among all of the libraries (Table 3), we observed the highest number of genes (3,878) were up-regulated in perisperm at 150 DAF compared with 60 DAF. Perisperm at 30 DAF also exhibited a high number of differentially expressed genes, with 2,115 genes up-regulated compared with flowers and 2,009 genes up-regulated compared with leaves. The library with the lowest amount of up-regulated unigenes (130) was perisperm at 60 DAF compared with leaves.

In contrast, the highest number of down-regulated genes (2,247) was detected in the perisperm at 90 DAF compared with 30 DAF. Perisperm in all sampling dates presented a high number of down-regulated genes compared with 30 DAF in a range of 1,241 to 2,247. The lowest number of down-regulated genes was observed in the perisperm at 60 DAF in relation to leaves (136), and this finding was similar to those obtained for up-regulated genes (Table 3).

The second DGE analysis was developed using only RNA-seq libraries from the perisperm at the five developmental stages. A total of 3,130 unigenes were down-regulated (Fig 1a), and 3,412 were up-regulated (Fig 1b). Compared to perisperm tissue at 30 DAF, 309 were down-regulated at 60 DAF, 557 at 90 DAF, 228 at 120 DAF, 88 at 150 DAF and some down-regulated genes overlapped in two or even three stages (Fig 1a). In contrast, 377 were specifically up-regulated at 60 DAF, 344 at 90 DAF, 932 at 120 DAF and 847 at 150 DAF (Fig 1b).

In addition, we annotated the top 10 unigenes exclusively expressed in each library. For this, we considered exclusively expressed unigenes that had an Interpro domain, RPKM >10 for one library and RPKM equal or less than two for all other libraries. Unigenes following these rules are summarized in S2 Table.

### Identification of putative transposable elements

We performed a BLAST analysis against a reference database of transposable elements (Repbase) [36] and identified 134 contigs with transposable elements (TE) fragments (S3 Table). From these contigs, 70 were annotated as class I TEs (52.24%) and 64 as class II (47.76%). These contigs were classified according to the following divisions: Gypsy (50), Copia (10), LINE (10), MuDR (29), Helitron (19), hAT (13), En/Spm (2), and Harbinger (1) (Table 4).

**Table 4:** Transcriptionally active transposable elements in *C*. *arabica* transcriptome.

Number of Contigs	TE Name	TE Class
50	Gypsy	I
10	Copia	I
10	LINE	I
29	MuDR	II
19	Helitron	II
13	hAT	II
2	EnSPM	II
1	Harbinger	II

### Identification of putative miRNA targets

miRNA identification using RNA-seq requires the construction of a special library. Therefore, the identification of mature miRNAs is beyond the scope of this study. However, transcripts that are regulated by miRNAs should contain sequences with almost perfect complementarity to known miRNAs. In plants, most miRNAs are encoded by gene families, and mature miRNAs typically have several target genes with similar complementary motifs in their mRNAs among several species [47].

In this study, we identified a total of 23,939 transcript targets on *C*. *arabica* transcriptome (S4 Table) regulated by 3,583 miRNA families. Among the miRNA families with putative targets in coffee transcriptome 3,068 (85.63%) have more than one target. These targets are mostly associated with miRNAs mir5658, mir5021 and mir414, which are typically overrepresented, given the massively amplified trinucleotide repeats (UGA, GAA, and UCA) in the mature sequences [48].

### Annotation of RFOs biosynthesis genes

We identified nine unigenes related to the biosynthesis of RFOs in our annotation (Table 5). The galactinol synthase (*GolS*), raffinose synthase (*RS*) and stacchyose synthase (*STS*) genes were selected for further analysis (Table 5). For each Arabica RFO-related unigene, we identified its ortholog in *Arabidopsis thaliana* and its respective first hit in *C*. *arabica* EST assemblies [5–6] and the *C*. *canephora* genome [3]. The Blast2GO annotation process (Table 5) allowed us to identify the conserved domains for RFO-related genes using Pfam database [49] (Table 5). In addition, galactinol, raffinose and stacchyose synthase candidate genes were mapped on the RFO metabolic pathway (galactose metabolism; MAP00052) available in the KEGG database (S4 Fig).

**Table 5:** Raffinose family oligosaccharide candidate genes.

Gene name	Enzymatic activity	TAIR database	*C*. *canephora* genome	CDD database	Pfam Entry	Protein length
*CaGolS2*	galactinol synthase	At1G56600	Cc03_g00450	PLN00176	pfam01501	345 aa
*CaGolS3*	galactinol synthase	At1G09350	Cc02_g35350	PLN00176	pfam01501	335 aa
*CaGolS4*	galactinol synthase	At1G60470	Cc11_g15250	PLN00176	pfam01501	339 aa
*CaGolS8*	galactinol synthase	At3G28340	Cc11_g14010	PLN00176	pfam01501	389 aa
*CaGolS9*	galactinol synthase	At3G06260	Cc11_g10580	PLN00176	pfam01501	350 aa
*CaRS1*	raffinose synthase	At1G55740	Cc05_g15530	PLN02355	pfam05695	678 aa
*CaRS5*	raffinose synthase	At5G40390	Cc07_g01840	PLN02355	pfam05695	782 aa
*CaRS6*	raffinose synthase	At5G20250	Cc06_g08070	PLN02355	pfam05695	870 aa
*CaSTS*	stachyose synthase	At4G01970	Cc01_g21600	PLN02355	pfam05695	879 aa

**Fig 2:**
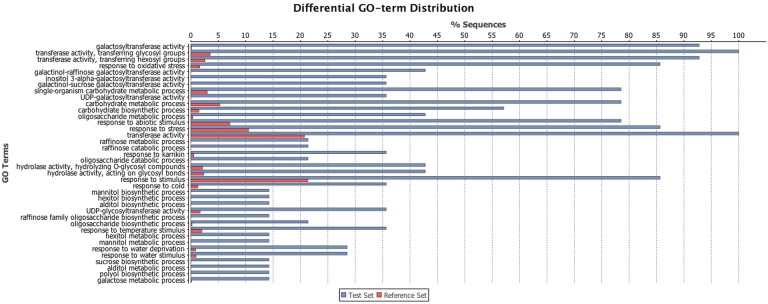
GO term categorization analysis performed by Blast2GO tools for RFO biosynthetic genes. GO categories annotated for RFOs candidate genes are indicated in blue; the global dataset, used as a reference, is indicated in red. GO categorization analyses were performed using the Blast2GO software with the default parameters.

GO categorization analysis was performed to identify functional categories related to RFO-biosynthesis (Fig 2). Among these transcripts, the most informative categories annotated for molecular function (S5 Fig) were galactosyltransferase activity (GO:008378), galactinol-raffinose galactosyltransferase activity (GO:0047268), galactinol-sucrose galactosyltransferase activity (GO:0047274), UDP-galactosyltransferase activity (GO:0035250) and inositol-3-alpha-galactosyltransferase activity (GO:0047216). For biological process (S6 Fig) the most representative functions were carbohydrate biosynthetic and metabolic process (GO:0016051), response to oxidative stress (GO:0006979), oligosaccharide biosynthetic process (GO:0009312), raffinose family oligosaccharide biosynthetic process (GO:0010325), raffinose metabolic and catabolic process (GO:0033530; GO:0034484), mannitol and sucrose biosynthetic process (GO:0019593; GO:0005986), response to abiotic stimulus (GO:0009628), response to cold (GO:0009409), response to oxidative stress (GO:006979) and response to water stress deprivation (GO:0009414).

### RFOs biosynthesis gene transcriptional profiles: Differential gene expression profiles among coffee tissues

The DGE profiles of the RFO-related genes were based on the RPKM values. We compared the expression data (RPKM values) available from the *C*. *canephora* genome hub [50] to our transcriptome gene expression profile, focusing on leaves and perisperm (average RPKM among all developmental stages).

**Fig 3:**
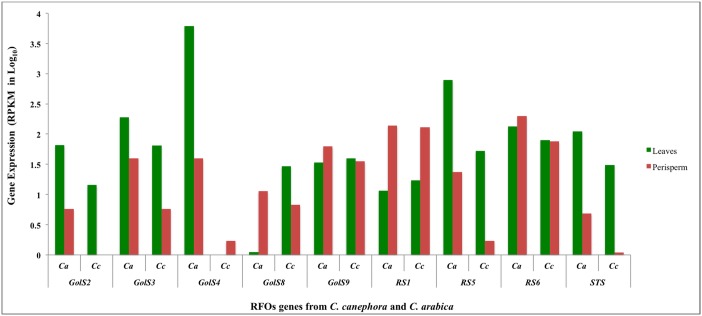
DGE comparison of raffinose biosynthesis-related genes in leaves and perisperm between *C*. *arabica* and *C*. *canephora*. RPKM values are represented in Log_10_ scale. Leaves noted in green, and perisperm in red. *Ca* = *C*. *arabica*. *Cc* = *C*. *canephora*. *C*. *canephora* RPKM values were obtained from the Coffee Genome Hub database [50].

We observed higher RPKM values in leaves than in the perisperm tissues for *GolS2*, *GolS3*, *RS5* and *STS* genes in both *Coffea* species. In contrast, we observed a high expression of *RS1* in the perisperm compared with leaves for both species. *CaGolS4* were highly expressed in leaves compared to perisperm in *C*. *arabica*, in opposition to what was observed in *C*. *canephora* (*CcGolS4*). Similar expression profiles were obtained for *GolS8* in perisperm for both species; however, in leaves, a higher expression was detected in *C*. *canephora* (*CcGolS8*) compared with *C*. *arabica* (*CaGolS8*). *GolS9* and *RS6* exhibited similar expression profiles in both coffee species, with little differences between leaves and perisperm (Fig 3).

### RFOs biosynthesis genes transcriptional validation

**Fig 4:**
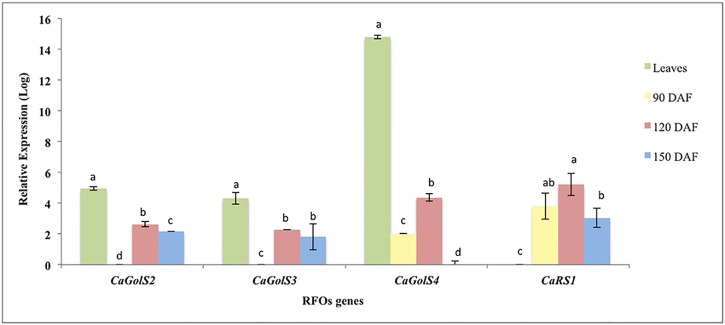
qRT-PCR analysis of selected RFO-related genes. Leaves are represented in green and perisperm in yellow (90 DAF), red (120 DAF) and blue (120 DAF). Relative expression values are represented in Log10 scale. Calibrator tissue is always the minimal gene expression value. Lower-case letters, from *a* to *d*, represent statistically significant differences for each RFO gene among coffee tissues (leaves and perisperm from 90 to 150 DAF).

To validate the DGE profile of the RNA-seq data, we chose four genes: *CaGolS2*, *CaGolS3*, *CaGolS4* and *CaRS1*. The qRT-PCR results were similar to those predicted by *in silico* expression for all RFO genes (Fig 4). *GolS* genes were more expressed in leaves than in the perisperm in all evaluated stages. The opposite pattern was observed for *CaRS1* gene, where the expression was up-regulated in the perisperm at any development stage compared with leaves.

## Discussion

### Assembly and functional annotation of C. arabica transcriptome

This report represents the first overview of *C*. *arabica* transcriptome gene atlas for flowers and perisperm during the initial development of fruits using RNA-seq. Most transcriptome studies on coffee have focused on the mature fruit at the last maturation stage, when they are ready to be collected and processed [4–6], or on leaves [8]. However, most chemical compounds of coffee grain are produced at the beginning of fruit development, when the perisperm is the predominant tissue. Its development can influence the grain size and chemical content of the final product [15–16] that consequently can influence coffee quality.

By comparing Arabica EST unigene sequences (35,153) with our transcript dataset (65,364), we identified 39,304 Arabica *no hit* sequences. This finding opens the possibility of identifying uncataloged new transcripts and rare or specific genes in the coffee transcriptome. Three possible explanations may account for this high number of *no hits*: i) Illumina technology improves the chance to identify rare transcripts and new gene isoforms [51]; ii) we used, for the first time, Arabica flowers and fruits during their initial development, which are not well represented in CafEST assembly; and iii) *de novo* transcriptome assembly using RNA-seq *single-end* technique typically generates a high number of unigenes [52].

Despite those differences, other studies in coffee obtained similar results in the annotation process, where *V*. *vinifera* sequences were the most similar organism to coffee protein sequences [3, 6, 28]. Also, the conserved domains and gene ontology results were similar to those found in other large-scale trancriptome analyses, where catalytic protein, kinases, cytochrome P450 and binding sites domains were the most frequently identified categories [28, 53].

### Transposable elements and miRNA targets in coffee transcriptome

Most of the TE-containing unigenes found in this transcriptome analysis (52.24%) were classified as LTR-retrotransposons, thus reinforcing the prevalence of this group of TE in the coffee transcriptome, as observed by Lopes et al. (2008) [13]. In our *de novo* trancriptome, the *Gypsy* superfamily of retrotransposons was the most abundant TE group.

miRNAs are small regulatory RNAs that play crucial roles in diverse aspects of plant development [54]. Identifying miRNA target genes is a fundamental step in determining the biological function for miRNAs. Families with a large number of targets may represent major hubs in gene regulatory networks, whereas those with fewer targets may act on specialized pathways. After excluding overrepresented families, the three *A*. *thaliana* miRNA families with the most putative targets are ath-miR854a, ath-miR834 and ath-miR838. mir854 is a highly conserved miRNA family, and its expression is predominant in flowers [55], which suggests that regulation of its targets may occur in coffee flowers. In contrast, mir834 is considered a “young” miRNA family [56, 57] that is involved in translation repression with low expression [58]. Mir838 regulates Dicer proteins as a intronic miRNA [59]. In summary, all 3 miRNAs that have several targets in coffee plants represent well-conserved families.

### Raffinose biosynthesis-related genes: Annotation and transcriptional analyses

In our transcriptome data, we identified five full-length genes *CaGolS*, three *CaRFS* and one *CaSTS* (Table 5). In addition, all RFOs genes were identified at least in one locus of the *C*. *canephora* genome [3], a *C*. *arabica* ancestor.

GO terms identification and conserved domain characterization were performed using all nine RFO genes to determine their putative molecular function and biological process (Fig 2; S5 and S6 Figs). The results corroborate the previously described functions for RFO genes because these genes were previously characterized as osmoprotectants and were up-regulated under water deficit, high-salinity soils, cold and heat stress conditions [17, 18, 19, 20, 60, 61].

Moreover, we compared the DGEs patterns of RFOs between our data (*C*. *arabica*) with those of *C*. *canephora* [3] (Fig 3). Most RFO biosynthesis genes (*CaGolS2*, *CaGolS3*, *CaRS1*, *CaRS5* and *CaSTS*) followed the same pattern in both *Coffea* species. *CaGolS2*, *CaGolS3*, *CaRS5* and *CaSTS* were higher expressed in leaves than in fruits, and only *CaRS1* was more expressed in fruits than in leaves.

However, the opposite result was obtained for the following four RFO genes: *CaGolS4*, *CaGolS8*, *CaGolS9* and *CaRS6*. One possible explanation for this result is the fact that *C*. *arabica* is the result of a recent natural hybridization between *C*. *canephora* and *C*. *eugenioides* [62]. Therefore, *C*. *arabica* could be preferentially expressing these four RFO genes from *C*. *eugenioides* subgenome (CaCe) instead of those from *C*. *canephora* (CaCc), as previously described for the citric acid cycle [63] and mannitol biosynthesis [64].

Our results for *CaGolS2*, *CaGolS3* and *CaGolS4* were similar to those of previous studies that described an up-regulation of these genes in leaves [13]. These genes were also up-regulated in the intermediary stages of fruit development and down-regulated at the initial stages of fruit development (perisperm) [17, 18]. Raffinose and stachyose oligosaccharides accumulated only transiently during coffee endosperm development [18]. In this context, we observed, as expected, low levels of transcriptional activity from these genes in the initial stages of the fruit maturation process (perisperm) since they are accumulated in the next stages during endosperm formation.

Genes related to RFO biosynthesis had their transcriptional levels validated using qRT-PCR analysis (Fig 4), thus reinforcing that our *in silico* analysis based on the RPKM values is reliable for transcriptional inferences.

## Conclusions

To our knowledge, this is the first large-scale trancriptome analysis of leaves, flowers and fruits during initial developmental stages in *C*. *arabica* using RNA-seq methodology. Our data have revealed TEs, miRNAs, new putative genes, larger number of full-length gene sequences and specific genes for the different tissues and fruit development stages. We provide a robust dataset for future transcriptome studies focused on the genetic mechanisms that can regulate fruit development and biosynthesis of coffee chemical compounds. This novel transcriptome survey provides a platform for future in-depth studies on numerous important metabolic pathways and will allow us to identify transcriptionally active genes in coffee tissues that are important for both coffee production and beverage quality.

## Ethical Standards

The experiments in this manuscript comply with the current laws of the country in which they were performed.

## Supporting Information

S1 Fig Coffee unigenes length distribution. (TIFF)

S2 Fig GO terms annotated for coffee transcripts. GO level categorization according to Blast2GO analysis: cellular component (green), molecular function (red) and biological process (blue).
(TIF)

S3 Fig Most represented Interpro domains associated with coffee unigenes. The total number found for each term is presented.
(TIFF)

S4 Fig Galactinol and Raffinose biosynthesis pathway (based on KEGG map 00052; Kanehisa et al., 2000). (TIFF)

S5 Fig GO term annotation associated with molecular function performed by Blast2GO tools for RFO biosynthetic genes. GO categories annotated for RFOS candidate genes are indicated in blue.
(TIFF)

S6 Fig GO term annotation associated with biological process performed by Blast2GO tools for raffinose genes. GO categories annotated for RFOS candidate genes are indicated in red.
(TIFF)

S1 Table Primer sequences used to for quantitative PCR analysis. (DOCX)

S2 Table TOP 10 unigenes exclusively expressed for each *C*. *arabica* transcriptome library. (XLSX)

S3 Table Transposable elements in *C*. *arabica* transcriptome. (DOCX)

S4 Table Transcript target on *C*. *arabica* transcriptome. (XLSX)
