# Supplementary figures and images for: Transcriptome Analysis of Leaves, Flowers and Fruits Perisperm of Coffea arabica L. Reveals the Differential Expression of Genes Involved in Raffinose Biosynthesis
Source: PLoS One. 2017 Jan 9;12(1):e0169595. doi: 10.1371/journal.pone.0169595 (PMC5221826; doi:10.1371/journal.pone.0169595)

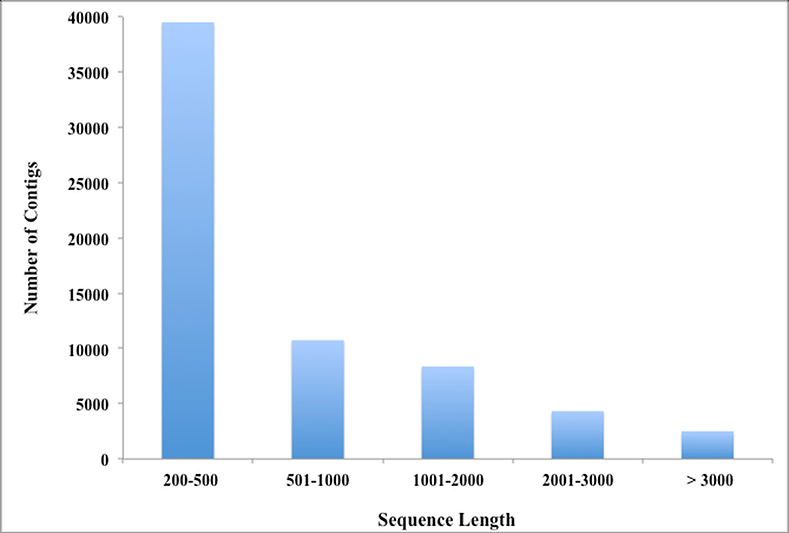

Supplement: S1 Fig — (TIFF) [file pone.0169595.s001.tiff]

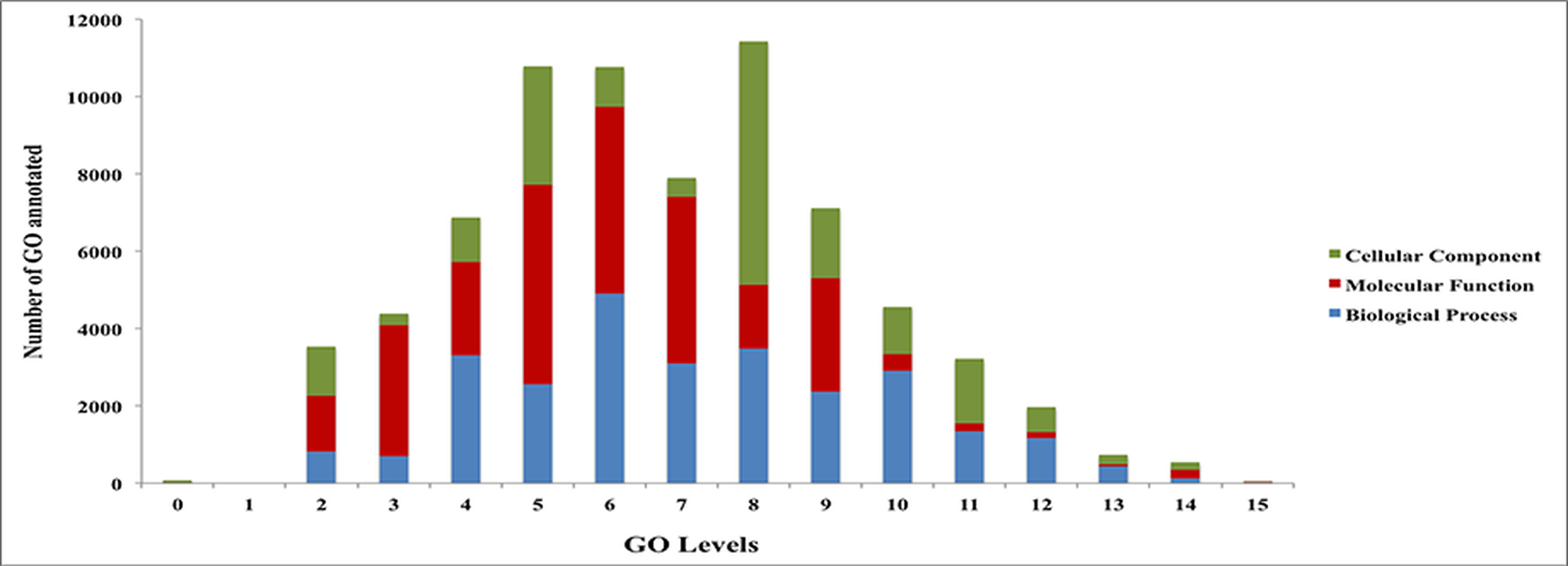

Supplement: S2 Fig — GO level categorization according to Blast2GO analysis: cellular component (green), molecular function (red) and biological process (blue). (TIF) [file pone.0169595.s002.tif]

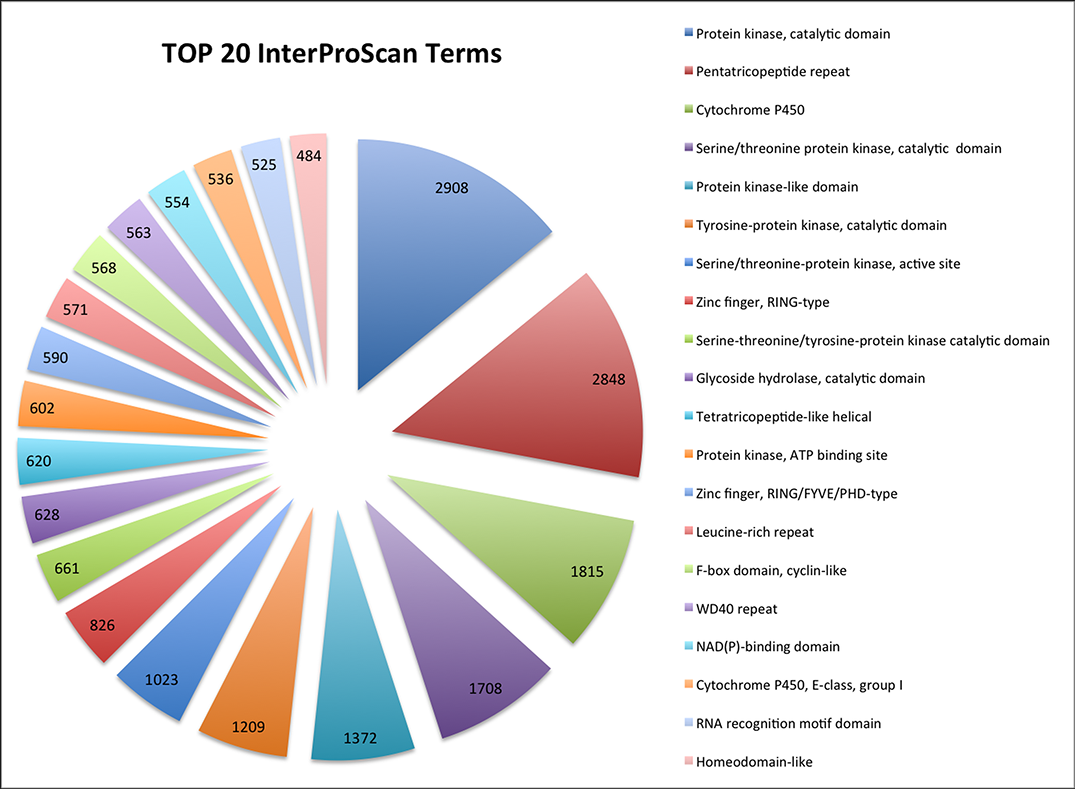

Supplement: S3 Fig — The total number found for each term is presented. (TIFF) [file pone.0169595.s003.tiff]

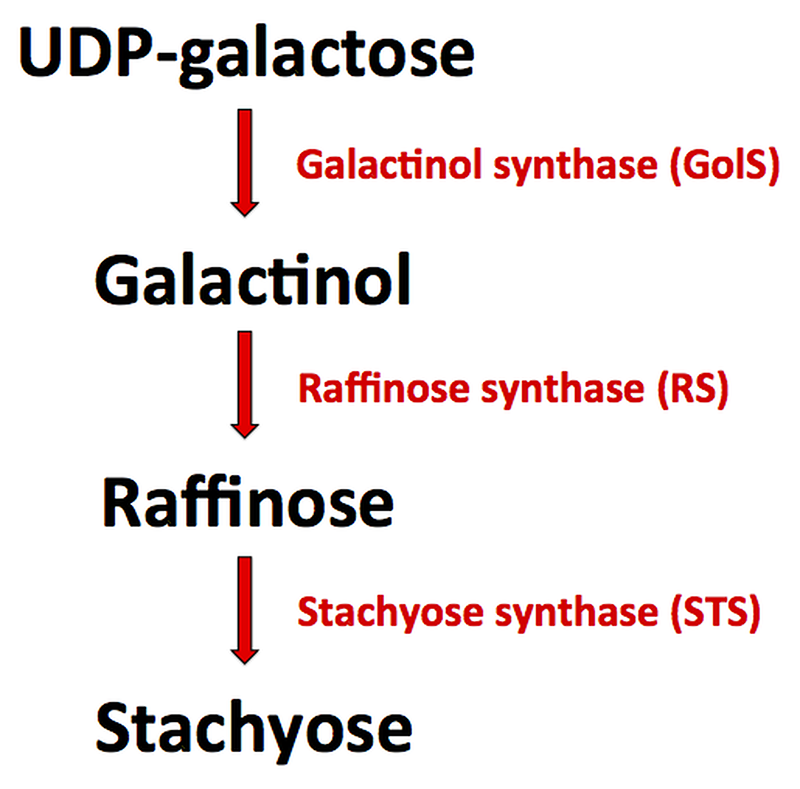

Supplement: S4 Fig — (TIFF) [file pone.0169595.s004.tiff]

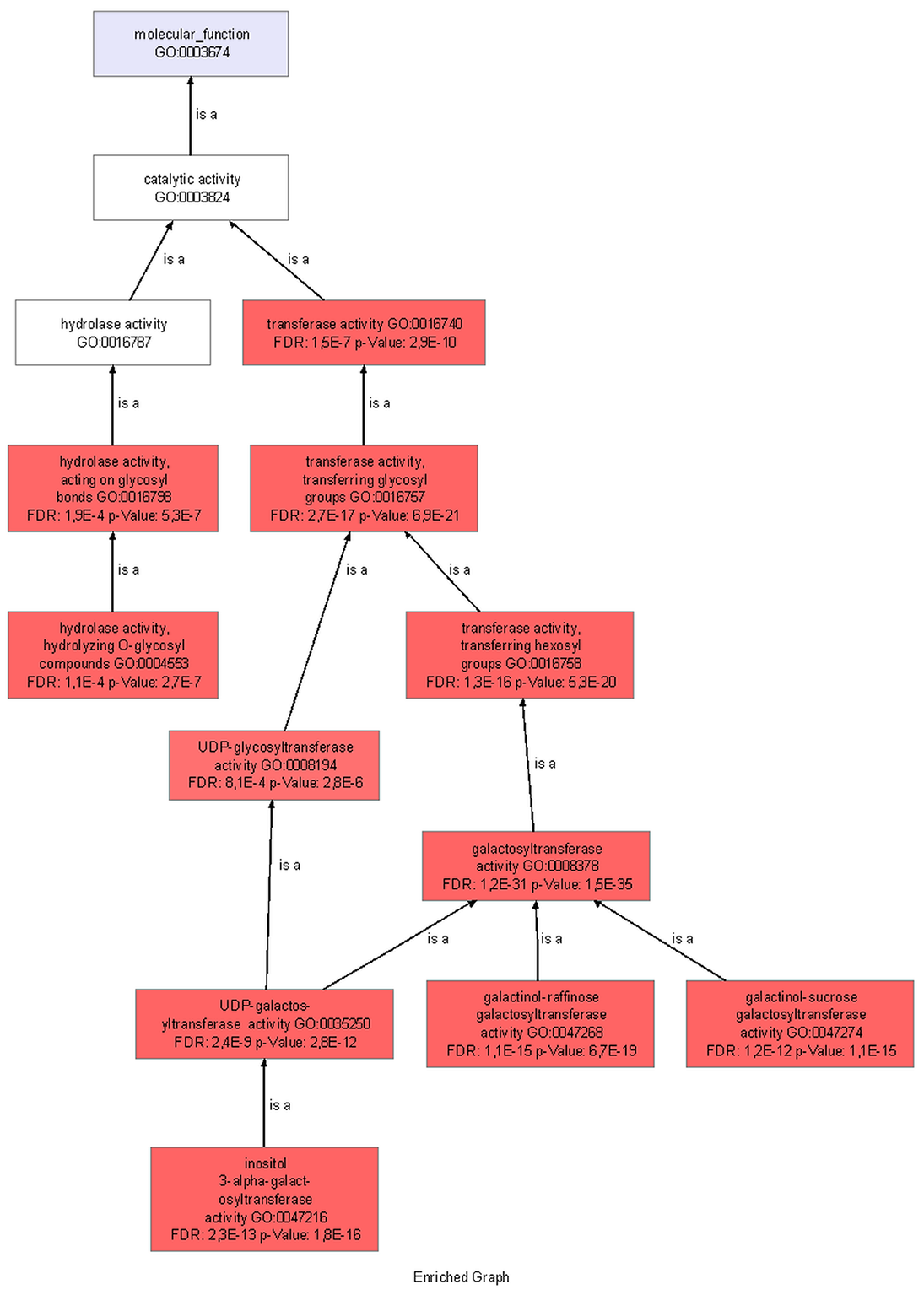

Supplement: S5 Fig — GO categories annotated for RFOS candidate genes are indicated in blue. (TIFF) [file pone.0169595.s005.tiff]

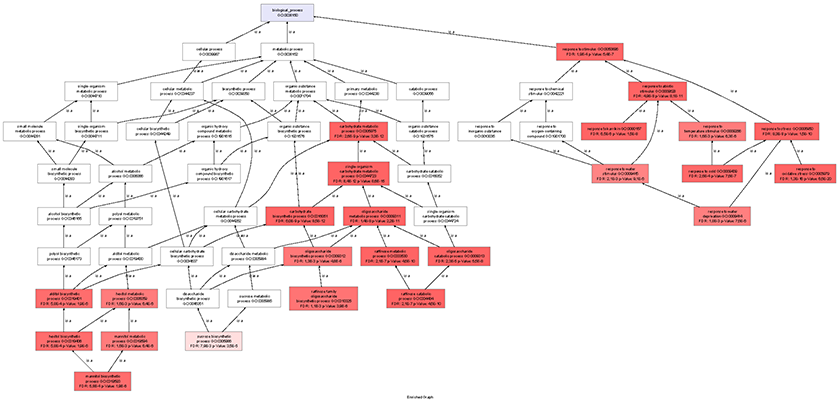

Supplement: S6 Fig — GO categories annotated for RFOS candidate genes are indicated in red. (TIFF) [file pone.0169595.s006.tiff]
