# Supplementary material for: Transcriptome Analysis of Leaves, Flowers and Fruits Perisperm of Coffea arabica L. Reveals the Differential Expression of Genes Involved in Raffinose Biosynthesis
Source: PLoS One. 2017 Jan 9;12(1):e0169595. doi: 10.1371/journal.pone.0169595 (PMC5221826; doi:10.1371/journal.pone.0169595)
Supplement: S1 Table — (DOCX) [file pone.0169595.s007.docx]

**S1 Table. Primer sequences used to for quantitative PCR analysis**

| **Gene Name** | **Primer Forward** | **Primer Reverse** | **Average efficiency** | **Amplicon size** |
| --- | --- | --- | --- | --- |
| *CaGolS2* | ATGGGATCGATGGAAATGAACTT | ACTTCCTGCCAAGAATGTCAC | 100% | 102 bp |
| *CaGolS3* | ATGGCTCCTGATACCGTTAG | ACCGTTCCCTGCCAGAAAT | 97% | 102 bp |
| *CaGolS4* | ATGGCCCCTCAAGAAGTAC | AGCTAAAAATGTAACGTACGCTC | 100% | 105 bp |
| *CaRS1* | ATGACTCAGAGAATGGGGAC | CACCGCCATCTTCACCAAAA | 98% | 103 bp |
| *CaEF1* | CTGTCCTTGATTGCCACACTTCT | CTTGGGCTCCTTCTCAAGCTC | 98% | 101 bp |
| *CaGAPDH* | AGGCTGTTGGGAAAGTTCTTC | ACTGTTGGAACTCGGAATGC | 100% | 70 bp |
