# Supplementary material for: Transcriptome Analysis of Leaves, Flowers and Fruits Perisperm of Coffea arabica L. Reveals the Differential Expression of Genes Involved in Raffinose Biosynthesis
Source: PLoS One. 2017 Jan 9;12(1):e0169595. doi: 10.1371/journal.pone.0169595 (PMC5221826; doi:10.1371/journal.pone.0169595)
Supplement: S3 Table — (DOCX) [file pone.0169595.s009.docx]

**S3 Table. Transposable elements in *C. arabica* transcriptome**

| **QUERY** | **Transposable element classification** | **align** | **qstart** | **qend** | **subjstart** | **subjend** | **evalue** | **score** |
| --- | --- | --- | --- | --- | --- | --- | --- | --- |
| comp37464 | Gypsy-30_MLP-I_1p:ClassI:LTR | 1030 | 3762 | 730 | 845 | 1874 | 0E+00 | 1198 |
| comp14003 | Gypsy-20_ST-I_1p:ClassI:LTR | 999 | 3551 | 573 | 495 | 1487 | 0E+00 | 1145 |
| comp21995 | L1-18_STu_2p:ClassI:LINE | 993 | 3088 | 137 | 278 | 1264 | 0E+00 | 712 |
| comp363132 | Copia-7_CP-I_1p:ClassI:LTR | 992 | 3221 | 378 | 151 | 1114 | 0E+00 | 1053 |
| comp18913 | Copia-55_MLP-I_1p:ClassI:LTR | 988 | 145 | 2931 | 573 | 1526 | 0E+00 | 609 |
| comp33790 | Helitron-4_FV_1p:ClassII | 968 | 3113 | 393 | 30 | 964 | 6E-150 | 476 |
| comp36534 | Gypsy-8_PX-I_1p:ClassI:LTR | 940 | 175 | 2742 | 12 | 897 | 2E-74 | 267 |
| comp34811 | Gypsy-8_PX-I_1p:ClassI:LTR | 935 | 686 | 3394 | 41 | 896 | 6E-96 | 332 |
| comp36231 | Helitron-2_PTr_1p:ClassII | 933 | 532 | 3237 | 603 | 1420 | 1E-80 | 294 |
| comp32278 | Tvv1_I_1p#2:ClassI:LTR:Copia | 924 | 2649 | 1 | 1 | 919 | 0E+00 | 1065 |
| comp34386 | Gypsy-8_PX-I_1p:ClassI:LTR | 914 | 3422 | 921 | 37 | 902 | 1E-79 | 283 |
| comp36424 | Gypsy-8_PX-I_1p:ClassI:LTR | 912 | 3236 | 687 | 21 | 896 | 8E-88 | 306 |
| comp35717 | Gypsy-8_PX-I_1p:ClassI:LTR | 911 | 3351 | 778 | 80 | 896 | 3E-66 | 243 |
| comp37238 | Helitron-1_STu_1p:ClassII | 898 | 4492 | 1889 | 17 | 757 | 3E-98 | 338 |
| comp37482 | Gypsy-8_PX-I_1p:ClassI:LTR | 897 | 4949 | 2424 | 34 | 896 | 4E-90 | 317 |
| comp36197 | Gypsy-8_PX-I_1p:ClassI:LTR | 897 | 375 | 2801 | 41 | 867 | 9E-68 | 246 |
| comp36622 | Gypsy-8_PX-I_1p:ClassI:LTR | 895 | 3021 | 448 | 68 | 896 | 5E-79 | 280 |
| comp36701 | Gypsy-8_PX-I_1p:ClassI:LTR | 893 | 297 | 2813 | 37 | 896 | 1E-85 | 300 |
| comp37294 | Gypsy-8_PX-I_1p:ClassI:LTR | 890 | 243 | 2747 | 41 | 896 | 3E-117 | 390 |
| comp369089 | Gypsy-8_PX-I_1p:ClassI:LTR | 889 | 221 | 2722 | 44 | 910 | 4E-118 | 391 |
| comp35941 | Gypsy-8_PX-I_1p:ClassI:LTR | 888 | 613 | 3198 | 90 | 910 | 1E-121 | 403 |
| comp36569 | Gypsy-8_PX-I_1p:ClassI:LTR | 886 | 510 | 3056 | 26 | 896 | 6E-92 | 320 |
| comp36456 | Gypsy-8_PX-I_1p:ClassI:LTR | 885 | 3408 | 916 | 101 | 896 | 1E-81 | 290 |
| comp37040 | Helitron-2_PTr_1p:ClassII | 882 | 377 | 2929 | 604 | 1416 | 1E-105 | 369 |
| comp36981 | Gypsy-8_PX-I_1p:ClassI:LTR | 882 | 3815 | 1428 | 35 | 896 | 3E-101 | 348 |
| comp29520 | Gypsy-8_PX-I_1p:ClassI:LTR | 881 | 318 | 2723 | 67 | 896 | 1E-91 | 314 |
| comp33971 | Gypsy-8_PX-I_1p:ClassI:LTR | 879 | 3275 | 717 | 80 | 896 | 5E-85 | 299 |
| comp34176 | Gypsy-8_PX-I_1p:ClassI:LTR | 879 | 2922 | 547 | 36 | 854 | 2E-82 | 290 |
| comp30253 | Gypsy-8_PX-I_1p:ClassI:LTR | 878 | 3325 | 866 | 57 | 896 | 2E-97 | 334 |
| comp32916 | Gypsy-8_PX-I_1p:ClassI:LTR | 876 | 2927 | 474 | 66 | 896 | 6E-96 | 330 |
| comp29128 | Gypsy-8_PX-I_1p:ClassI:LTR | 873 | 3049 | 695 | 56 | 896 | 8E-60 | 222 |
| comp36890 | Gypsy-8_PX-I_1p:ClassI:LTR | 872 | 535 | 2979 | 35 | 827 | 1E-74 | 248 |
| comp32326 | Gypsy-8_PX-I_1p:ClassI:LTR | 871 | 688 | 2910 | 57 | 896 | 3E-73 | 264 |
| comp33402 | Gypsy-8_PX-I_1p:ClassI:LTR | 865 | 146 | 2587 | 77 | 896 | 1E-100 | 343 |
| comp33294 | Helitron-2_PTr_1p:ClassII | 861 | 3474 | 988 | 602 | 1402 | 3E-111 | 385 |
| comp37533 | Helitron-2_PTr_1p:ClassII | 855 | 534 | 3032 | 602 | 1389 | 8E-135 | 456 |
| comp35162 | Gypsy-8_PX-I_1p:ClassI:LTR | 852 | 705 | 3092 | 41 | 868 | 4E-79 | 281 |
| comp37481 | Helitron-2_PTr_1p:ClassII | 850 | 559 | 3054 | 618 | 1416 | 6E-118 | 407 |
| comp364326 | Gypsy-112_GM-I_1p:ClassI:LTR | 848 | 258 | 2681 | 69 | 866 | 5E-87 | 302 |
| comp15651 | Gypsy-8_PX-I_1p:ClassI:LTR | 846 | 2487 | 52 | 71 | 896 | 2E-95 | 324 |
| comp362012 | Helitron-2_PTr_1p:ClassII | 842 | 2349 | 16 | 618 | 1401 | 2E-108 | 372 |
| comp33146 | Gypsy-112_GM-I_1p:ClassI:LTR | 841 | 217 | 2643 | 69 | 833 | 5E-70 | 256 |
| comp30195 | Gypsy-8_PX-I_1p:ClassI:LTR | 838 | 3014 | 672 | 93 | 896 | 8E-72 | 260 |
| comp27987 | Helitron-2_PTr_1p:ClassII | 837 | 2599 | 254 | 609 | 1396 | 6E-55 | 208 |
| comp31858 | Gypsy-8_PX-I_1p:ClassI:LTR | 833 | 3076 | 962 | 52 | 873 | 1E-62 | 230 |
| comp36464 | Gypsy-8_PX-I_1p:ClassI:LTR | 831 | 1108 | 3273 | 95 | 896 | 4E-94 | 331 |
| comp36699 | Gypsy-8_PX-I_1p:ClassI:LTR | 817 | 5 | 2437 | 93 | 896 | 0E+00 | 612 |
| comp37266 | Gypsy-8_PX-I_1p:ClassI:LTR | 817 | 6443 | 4140 | 90 | 896 | 4E-123 | 415 |
| comp26902 | Gypsy-8_PX-I_1p:ClassI:LTR | 815 | 2747 | 531 | 161 | 896 | 6E-69 | 248 |
| comp37454 | Gypsy-8_PX-I_1p:ClassI:LTR | 813 | 1114 | 3357 | 136 | 896 | 2E-83 | 297 |
| comp20034 | EnSpm-6_VV_Transposase#2:ClassII | 808 | 3711 | 1300 | 159 | 963 | 0E+00 | 937 |
| comp23303 | Tad1-7_PGr_2p:ClassI:LINE:I | 792 | 2606 | 267 | 475 | 1252 | 7E-95 | 327 |
| comp34776 | MuDR-12_VV_Transposase:ClassII | 788 | 3263 | 948 | 12 | 741 | 2E-86 | 298 |
| comp27760 | Gypsy-8_PX-I_1p:ClassI:LTR | 780 | 44 | 2053 | 150 | 896 | 3E-68 | 245 |
| comp375321 | Helitron-2_PTr_1p:ClassII | 774 | 2289 | 82 | 598 | 1333 | 3E-93 | 323 |
| comp25193 | Helitron-2_PTr_1p:ClassII | 768 | 2260 | 239 | 674 | 1422 | 4E-76 | 274 |
| comp37531 | Copia-94_VV-I_1p:ClassI:LTR:Copia | 767 | 2 | 2287 | 616 | 1364 | 0E+00 | 946 |
| comp10406 | Gypsy-3_ST-I_1p:ClassI:LTR | 750 | 2635 | 413 | 83 | 832 | 0E+00 | 862 |
| comp26887 | Gypsy-8_PX-I_1p:ClassI:LTR | 743 | 2572 | 431 | 177 | 901 | 2E-122 | 396 |
| comp32271 | L1-5_Mad_2p:ClassI:LINE:L1 | 730 | 2220 | 100 | 806 | 1509 | 3E-104 | 364 |
| comp37504 | Gypsy-8_PX-I_1p:ClassI:LTR | 722 | 2715 | 745 | 220 | 912 | 1E-66 | 241 |
| comp33840 | hAT-7_VV_Transposase#2:ClassII:TIR | 708 | 846 | 2936 | 15 | 688 | 4E-134 | 430 |
| comp34996 | MuDR-13_VV_Transposase#2:ClassII | 700 | 2852 | 810 | 14 | 686 | 1E-94 | 322 |
| comp36288 | Copia-89_ST-I_1p:ClassI:LTR:Copia | 695 | 1116 | 3158 | 109 | 801 | 0E+00 | 909 |
| comp29368 | hAT-4_STu_1p:ClassII:TIR:hAT | 692 | 2692 | 644 | 10 | 670 | 2E-124 | 404 |
| comp353512 | MuDR-21_VV_Transposase:ClassII | 689 | 4102 | 2135 | 39 | 712 | 2E-156 | 498 |
| comp364410 | hAT-1_GM_1p:ClassII:TIR:hAT | 687 | 1320 | 3287 | 39 | 713 | 5E-172 | 527 |
| comp304812 | hAT-1_GM_1p:ClassII:TIR:hAT | 674 | 2330 | 399 | 48 | 707 | 0E+00 | 569 |
| comp26943 | hAT-7_VV_Transposase#2:ClassII | 673 | 107 | 2080 | 11 | 679 | 6E-111 | 365 |
| comp373571 | Copia-56_VV-I_2p:ClassI:LTR:Copia | 670 | 1992 | 1 | 3 | 659 | 0E+00 | 964 |
| comp34040 | MuDR-5_FV_1p:ClassII | 665 | 2873 | 1047 | 73 | 710 | 2E-110 | 372 |
| comp33615 | hAT-6_VV_Transposase#2:ClassII | 657 | 235 | 2196 | 33 | 652 | 5E-162 | 490 |
| comp37470 | Gypsy-112_GM-I_1p:ClassI:LTR | 655 | 572 | 2527 | 269 | 864 | 3E-108 | 361 |
| comp33513 | hAT-6_VV_Transposase#2:ClassII | 654 | 219 | 2177 | 30 | 648 | 4E-139 | 436 |
| comp33775 | MuDR-5_FV_1p:ClassII | 652 | 2857 | 1025 | 73 | 719 | 2E-102 | 345 |
| comp19698 | Gypsy-8_PX-I_1p:ClassI:LTR | 652 | 2519 | 744 | 274 | 896 | 2E-73 | 260 |
| comp36875 | MuDR-5_FV_1p:ClassII | 646 | 399 | 2261 | 76 | 715 | 7E-136 | 436 |
| comp35772 | hAT-6_VV_Transposase#2:ClassII | 646 | 489 | 2402 | 32 | 642 | 3E-107 | 352 |
| comp30874 | hAT-6_VV_Transposase#2:ClassII | 638 | 252 | 2162 | 32 | 642 | 1E-110 | 356 |
| comp34867 | MuDR-5_FV_1p:ClassII | 632 | 2453 | 621 | 172 | 798 | 8E-94 | 321 |
| comp33492 | Helitron-2_PTr_1p:ClassII | 625 | 5 | 1798 | 848 | 1422 | 1E-70 | 258 |
| comp37498 | Helitron-2_PTr_1p:ClassII | 623 | 2735 | 948 | 853 | 1416 | 1E-61 | 234 |
| comp35239 | Helitron-4_FV_1p:ClassII | 618 | 110 | 1744 | 25 | 622 | 9E-80 | 228 |
| comp34562 | MuDR-21_VV_Transposase:ClassII | 616 | 396 | 2195 | 10 | 604 | 2E-73 | 260 |
| comp32183 | Gypsy-8_PX-I_1p:ClassI:LTR | 600 | 2 | 1774 | 40 | 583 | 2E-85 | 301 |
| comp33245 | MuDR-16_ALy_1p:ClassII | 598 | 437 | 2191 | 38 | 607 | 1E-71 | 255 |
| comp21417 | Helitron-1B_SBi_1p:ClassII | 595 | 1763 | 15 | 235 | 820 | 5E-150 | 482 |
| comp11878 | L1-6_ALy_2p:ClassI:LINE:L1 | 591 | 2389 | 638 | 605 | 1182 | 1E-119 | 370 |
| comp34158 | MuDR-4_VV_Transposase#2:ClassII | 591 | 989 | 2755 | 238 | 814 | 2E-91 | 312 |
| comp34885 | MuDR-12_VV_Transposase:ClassII | 583 | 2071 | 3786 | 161 | 741 | 6E-78 | 275 |
| comp30532 | MuDR-4_VV_Transposase#2:ClassII | 579 | 382 | 2100 | 247 | 811 | 3E-94 | 316 |
| comp26560 | MuDR-7_Mad_1p:ClassII | 573 | 2683 | 968 | 84 | 643 | 5E-131 | 424 |
| comp34232 | MuDR-4_VV_Transposase#2:ClassII | 573 | 2036 | 324 | 256 | 814 | 2E-92 | 316 |
| comp35193 | hAT-7_VV_Transposase#2:ClassII:TIR | 568 | 2952 | 1336 | 11 | 567 | 3E-83 | 289 |
| comp26611 | MuDR-1_Mad_1p:ClassII | 567 | 538 | 2145 | 21 | 559 | 4E-65 | 231 |
| comp36938 | MuDR-5_FV_1p:ClassII | 566 | 4169 | 2481 | 163 | 722 | 2E-125 | 414 |
| comp28460 | Helitron-2_PTr_1p:ClassII | 563 | 2858 | 1248 | 911 | 1422 | 1E-75 | 272 |
| comp35367 | Gypsy-8_PX-I_1p:ClassI:LTR | 557 | 524 | 2107 | 30 | 535 | 1E-52 | 200 |
| comp34471 | MuDR-5_FV_1p:ClassII | 556 | 562 | 2190 | 175 | 705 | 2E-103 | 348 |
| comp35765 | Copia-94_VV-I_1p:ClassI:LTR:Copia | 550 | 272 | 1921 | 817 | 1365 | 0E+00 | 861 |
| comp34173 | MuDR-1_Mad_1p:ClassII | 550 | 946 | 2445 | 16 | 561 | 5E-84 | 286 |
| comp33539 | Gypsy-8_PX-I_1p:ClassI:LTR | 546 | 2133 | 613 | 394 | 896 | 3E-60 | 220 |
| comp31153 | EnSpm-1_STu_2p:ClassII:TIR:CACTA | 533 | 12 | 1589 | 117 | 642 | 0E+00 | 568 |
| comp36215 | Helitron-2_PTr_1p:ClassII | 532 | 65 | 1651 | 1 | 526 | 3E-103 | 357 |
| comp36507 | MuDR-1_STu_1p:ClassII | 525 | 1429 | 2952 | 43 | 549 | 8E-91 | 312 |
| comp334127 | MuDR-3_VV_Transposase:ClassII | 524 | 599 | 2164 | 212 | 728 | 3E-73 | 264 |
| comp25003 | L1-7_ZM_2p:ClassI:LINE:L1 | 496 | 2342 | 861 | 627 | 1117 | 9E-116 | 384 |
| comp12014 | L1-7_ZM_2p:ClassI:LINE:L1 | 486 | 2319 | 868 | 637 | 1117 | 2E-123 | 405 |
| comp33436 | Gypsy-8_PX-I_1p:ClassI:LTR | 480 | 2558 | 1131 | 445 | 911 | 5E-123 | 397 |
| comp32846 | MUMT_1p:ClassII | 478 | 657 | 2069 | 1 | 468 | 9E-105 | 338 |
| comp33073 | MUMT_1p:ClassII | 473 | 1625 | 219 | 1 | 467 | 1E-106 | 342 |
| comp30838 | MUMT_1p:ClassII | 461 | 1737 | 379 | 1 | 452 | 1E-107 | 346 |
| comp26747 | MUMT_1p:ClassII | 459 | 748 | 2097 | 1 | 451 | 9E-97 | 317 |
| comp342092 | HAT-10_Mad_1p:ClassII:TIR:hAT | 453 | 562 | 1899 | 69 | 520 | 4E-126 | 396 |
| comp30763 | MuDR-6_STu_1p:ClassII | 448 | 85 | 1416 | 352 | 796 | 3E-102 | 338 |
| comp372908 | MUMT_1p:ClassII | 448 | 1391 | 2704 | 29 | 473 | 3E-101 | 338 |
| comp26646 | MuDR-6_VV_Transposase:ClassII | 447 | 2272 | 1022 | 846 | 1289 | 2E-66 | 244 |
| comp36856 | MuDR-6_VV_Transposase:ClassII | 445 | 7048 | 5714 | 870 | 1283 | 4E-113 | 400 |
| comp25801 | Helitron-1_STu_1p:ClassII | 441 | 286 | 1572 | 17 | 421 | 2E-65 | 235 |
| comp37394 | TONT2-I_PV_1p:ClassI:LTR:Copia | 437 | 1335 | 40 | 11 | 443 | 8E-157 | 476 |
| comp35822 | Gypsy-43_Mad-I_3p:ClassI:LTR | 432 | 241 | 1464 | 215 | 637 | 1E-53 | 201 |
| comp37376 | hAT-7_VV_Transposase#2:ClassII:TIR | 411 | 1056 | 2279 | 153 | 558 | 2E-85 | 270 |
| comp31932 | L1-5_ALy_2p:ClassI:LINE:L1 | 375 | 3361 | 4443 | 571 | 931 | 4E-173 | 253 |
| comp29071 | L1-7_ALy_1p:ClassI:LINE:L1 | 363 | 640 | 1725 | 350 | 711 | 5E-158 | 347 |
| comp338792 | L1-18_STu_2p:ClassI:LINE:L1 | 343 | 3501 | 2488 | 646 | 988 | 6E-66 | 244 |
| comp371371 | Copia47-PTR_I_1p#2:ClassI:LTR:Copia | 313 | 5929 | 4997 | 249 | 561 | 9E-152 | 484 |
| comp30381 | Gypsy-112_GM-I_1p:ClassI:LTR | 300 | 2088 | 2984 | 597 | 889 | 3E-68 | 247 |
| comp33472 | MtPH_1p:ClassII:TIR:PIF-Harbinger | 283 | 1145 | 321 | 70 | 348 | 3E-59 | 209 |
| comp37427 | RAM14_I_MT_1p#2:ClassI:LTR | 271 | 951 | 145 | 1 | 271 | 1E-84 | 283 |
| comp28907 | Helitron-1_PTr_1p:ClassII | 270 | 2664 | 1954 | 231 | 500 | 6E-64 | 226 |
| comp373865 | Copia-33_GM-I_1p:ClassI:LTR:Copia | 258 | 2295 | 1525 | 159 | 401 | 7E-85 | 291 |
| comp50380 | L1-5_ALy_2p:ClassI:LINE:L1 | 255 | 3241 | 2477 | 436 | 690 | 2E-144 | 267 |
| comp31938 | MuDR-6_VV_Transposase:ClassII | 253 | 461 | 1219 | 865 | 1111 | 4E-56 | 212 |
| comp23343 | HAT-9_Mad_1p:ClassII:TIR:hAT | 209 | 1809 | 1186 | 222 | 430 | 3E-107 | 243 |
